# International Medical Congress

**Published:** 1887-08

**Authors:** 


### NINTH INTERNATIONAL MEDICAL CONGRESS.

The medical profession of the world will have an opportunity for interchanging views and comparing observations at the reunions to be held in Washington City, commencing on the 5th of September.

It is not generally understood that "the Congress will consist of such members of the regular medical profession as shall have registered and taken out their ticket of admission, and of such other scientific men as the Executive Committee of the Congress shall deem desirable to admit. The dues of membership for residents of the United States will be ten dollars ($10.00). There will be no dues for members residing in other countries. Each member will be entitled to receive a copy of the Transactions of the Congress when published by the Executive Committee." It will be noted that no delegates of State or local societies are recognized, but all members of the regular medical profession may receive tickets of admission upon paying ten dollars and entering their names upon the roll.

This liberal provision for membership in the Congress excludes none who come within the pale of the regular medical profession, and while the scientific work has been assigned for the most part, there will no doubt be an opportunity for presenting any voluntary paper which may be reported in due time to the chairman of the Executive Committee or to the Secretary-General of the Congress.

For the sake of method in conducting the business of the various sections, there will be a programme published at an early day embodying all the papers and discussions in the order of presentation, so that those having part in the work shall understand their allotment, while those desirous of attending any special section or hearing the paper of any particular person will be advised of the time and place of attendance.

Considering the progress which is being made annually in every department of medical science, and the array of talent amongst our own members and those from abroad, there will certainly be a greater flood of light thrown upon the pathway of the physician at this meeting of the Congress than has ever resulted from any previous assemblage of the medical profession in the history of the world.

We trust all may co-operate in securing the largest measure of success for the Congress.

### ITEMS.

DR. W. S. ARMSTRONG, of this city, is spending the summer at Porter Springs.

DR. C. A. BROOKS, of Americus, was married a few weeks since to Miss Willie Copeland.

Dr. D. W. HAMMOND, of Macon, ex-Vice-President of the Medical Association of Georgia, died at his home in Macon a few days ago.

PROF. V. H. TALIAFERRO will deliver the opening address of the Atlanta Medical College at the approaching session, which begins October 5th.

THE International Medical Congress, which meets in Washington, D. C., September 5, will no doubt be the largest gathering of physicians ever assembled in America.

DOES the City Council know that if the Benevolent Home had been paid seventy-five cents a day per capita for caring for the sick-poor of Atlanta last year, it would have made a net profit of $6,000?

THE indications are that there will be a large number of Georgia physicians in attendance upon the meeting of the International Medical Congress which meets in Washington, D. C., September 5.

IN our editorial of last month, on the cost of caring for the sick-poor of this city, we stated only such facts as can be substantiated by the best men in the city, and if these facts have hurt any one we cannot help it, and do not propose to try. As long as we conduct THE JOURNAL we expect to follow our convictions of right in expressing ourselves upon public matters, let it hurt whom it may.

AN ENTERPRISING DOCTOR.—There is said to be a practitioner in New York who promises to pay half the funeral expenses in all his unsuccessful cases. The device is ingenious, but not commendable.—*Press and Circular.*

J. W. AINGER, representing the well-known publisher of the *Eclectic* magazine, Mr. E. R. Pelton, 25 Bond street, N. Y., gave THE JOURNAL a pleasant call a few days ago. The *Eclectic* is one of the best literary magazines published.

A NEW DRUG FIRM.—Smith & Bradfield have purchased the stock of Pinson, Dozier & Co., and have now one of the handsomest drug stores in the city at 102 Whitehall street. In addition to a full line of drugs of other manufacturers, they will carry a complete stock of Parke, Davis & Co.'s goods.

UNIVERSITY OF PARIS.—There are altogether 10,679 students of the University of Paris, of which 3,696 are medical, 467 for the sciences, and 1,766 pharmaceutical. 168 of these students are females, and 108 of their number are studying medicine. The foreign medical students number 593. In the report of the General Council of Education, special mention is made of the zeal and success of the female students.—*Ex.*

AT the quarterly meeting of the Illinois State Board of Health, held July 8th, 1887, the following resolution, offered by Dr. Clark, of the board, was adopted : *Resolved*, That the phrase, "medical colleges in good standing," in the first section of the Act to Regulate the Practice of Medicine, approved June 16, 1887, is hereby defined to include only those colleges which shall, after the sessions of 1890–91, require four years of professional study, including any time spent with a preceptor, and three regular courses of lectures, as conditions of graduation, and shall otherwise conform to the Schedule of Minimum Requirements heretofore adopted by the board.

COMPENSATION FOR EXPERTS.—The majority of decisions, and in many States the law itself, favors the view that medical men should be adequately compensated for giving expert testimony. Indeed, in the present state of the matter it can be only ignorant or grossly partisan judges who refuse to allow such compensation, except, of course, in the States or Territories in which a law, like that passed by the stupid legislators of Indiana, exists. —*N. Y. Medical Record.*

THE PROGRESS OF HOMŒOPATHY.—Dr. F. H. Orme, of this city, President of the American Institute of Homœopathy, in his address before that body in Saratoga, a few weeks ago, stated that homœopathy was first introduced in America in 1825 by Dr. Gram. Since that time it shows the following growth: Practitioners, 11,000; medical colleges, 14; matriculates annually, 1,200; graduates annually, 400; hospitals (with 4,500 beds), 57; insane asylums, 3; dispensaries, 48; societies, 150; journals, 23; pharmacies, 33; colleges of specialties, 1. Thirty-four dispensaries report for one year, 1886, 142,629 patients provided for with 376,886 prescriptions.

THE NEW PRINCETON REVIEW for July presents a series of articles which possess in an unusual degree the quality of being interesting; the subjects discussed are not only varied but entertaining. Mr. R. W. Gilder, the editor of the *Century Magazine*, emphasizes "Certain Tendencies in Current Literature," and in. terprets them as indications of the thirst for life and reality in art- Mr. S. G. W. Benjamin outlines the development of "American Art Since the Centennial," and enumerates the multiplying indications of original and distinctively American evolution of art in this country. "The Theory of Prohibition" is examined at length and in a thoroughly candid spirit by Mr. Sanford H. Cobb, who discriminates sharply between the true and the false grounds on which it is based in current discussion.

THE JOURNAL AND THE ASSOCIATION.—We notice that the *Journal of the American Medical Association* is being published as THE JOURNAL of the American Medical Association. We trust that this does not indicate the editor's opinion of the relative importance of the Journal and the Association. If it does, we do not wonder that the paper on which the title-page is printed has blushed a vivid pink.—*Medical and Surgical Reporter.*

PROFESSOR SHALER'S article on tornadoes and cyclones, in *Scribner's Magazine* for August, contains reproductions of two instantaneous photographs of a tornado which passed over Jamestown, Dakota, on June 6, 1887. The publishers had made a special search for negatives of storms, and given notice of it to many Western photographers. This fortunate opportunity occurred after the article was already in type. It is believed that a complete cloud-whirl has not before been photographed; at all events, thorough inquiry has discovered no instance.

HABITUAL CONSTIPATION IN CHILDREN.—"We know that infant feeding is the subject of great difference of opinion among mothers and nurses, and sometimes children thrive on a diet which doctors consider unsuitable. There is no doubt, I think, that some malted foods added to milk are both digestible and nourishing; they stimulate the bowels to regular action, the motions becoming normal in appearance and quantity, whilst body-growth increases at a proper rate. I have known of many cases where Mellin's food (which has now become a very popular preparation) has agreed well with young children, improving their nutrition in a remarkable manner. It is soluble in cold water, and said to contain 87 per cent. of dextrin, maltose, etc., but the efficacy of the food, according to Mr. G. Wigner, consists in its containing those nitrogenous and phosphatic principles which contribute to the growth of bone and tissue. The starchy and sugar elements are in small proportion. When mixed with milk its digestive power is increased, and is most valuable as a nutritious food. In June, 1884, I saw a child eight months old who was brought up by hand, and did not thrive owing to flatulent distention of the bowels and constipation, the motions being hard and pebbly with much straining in defecation. Mellin's food, mixed with milk, quickly overcame these troubles, and the child soon became strong and healthy. In another case, a child, aged one year, also brought up by hand, was very constipated, thin and whining, with the wrinkled face of abdominal trouble. It was rapidly passing from bad to worse. From the day Mellin's food was given an improvement set in, the sickness ceased, the abdomen became soft, and the action of the bowels was healthy and regular."—Dr. William H. Day, in *London Medical Press*, May 11th, 1887.

DIARRHŒA OF CHILDREN.—As the season of the year approaches in which diarrhœas are especially fatal in children, we feel it not improper to call the attention of our readers to a remedy which, though used by some practitioners, is, we think, still neglected by many of the profession. We refer to the phosphate of sodium. In the summer diarrhœas connected with a lack of digestive power, in which the stools are either clay-colored or habitually greenish, phosphate of sodium often brings a favorable response when the ordinary remedy for diarrhœa seems to irritate rather than do good. In nursing children it may be given in the milk, 10 grains of it in each bottle, or it may be given after eating, dissolved in a little water. It should be administered always in repeated small doses and not in a single large dose. Where there is habitual constipation, with occasional attacks of diarrhœa in young children, it is especially serviceable. It probably has some distinct specific action upon the glandular organs of the intestinal tract.—*Therapeutic Gazette.*

THE DISCOVERER OF ANÆSTHESIA.—The diatribe of the *Northwestern Lancet* of July 15th, in regard to the discoverer of anæsthesia, indicates that the editor does not possess normal visual organs, nor is he myopic or presbyopic, but is laboring under a progressive form of cataract, or that disorder of the vitreous body, giving origin to muscæ volitantes. One thing is patent from his editorial, that his vision is seriously disordered, and that he cannot discern objects which others discern clearly. In the light of the Argand lamp which Sims brought to bear upon the discovery of ether as an anæsthetic by Crawford W. Long, it is so clear that he who runs may read his title to this distinction. The balderdash of Sidney Smith's aphorism, which he quotes, has no application to the case, as Long actually put into practice the great principle involved in the application of ether for annulling the suffering from surgical operations before any other had proposed or even dreamed of this mode of effecting anæsthesia.

PRECOCIOUS GUMMATA.—The close and widespread study of syphilis within the past fifteen years has conclusively shown that the old and dogmatic division of the disease into three sharply-marked periods must soon be very much modified, or perhaps even discarded, and that, although the terms primary, secondary and tertiary, as applied to stages of syphilis, present the advantage of clearness and simplicity in study and description, and may even be clinically true as regards a large number of cases, yet there are very many in which such a division is inappropriate, since we observe in some the so-called tertiary lesions in the secondary period; in others seemingly secondary lesions in the tertiary period, and perhaps co-existing with well-marked lesions of that period; or, again, cases of tertiary lesions concomitant with secondary lesions. To hold, then, that superficial lesions belong to and are only found in the early or secondary period, and that they are followed later on by lesions involving the tissues more profoundly, is in reality to sacrifice facts for simplicity of description.

Though there exist comprehensive descriptions of precocious nervous, osseous, articular, ocular and superficial ulcerative dermal affections due to syphilis, a systematic description of the clinical history of precocious gummata is wanting. In *The American Journal of the Medical Sciences* for July, Dr. Robert W. Taylor presents the clinical histories of a selected number of his cases, from which he traces a clear and satisfactory description of these not common nor yet infrequent eruptions. His studies convince him that there are three forms of the precocious gummata: The first, the generalized form; the second, the localized form; and the third, the neurotic form, which in some of its features resembles erythema nodosum. Of each of these three forms, moreover, there are two varieties: a resolutive, or non-ulcerative, and an ulcerative variety.

The clinical history of the generalized form of precocious syphilide is as follows: As early as the eighth week of the infection the patient notices either small circumscribed swellings under the skin generally unattended with pain, and only perceptible to the touch, or this stage may escape him, and his attention is at first arrested by a number of bright red spots. These gummata are found to be round tumors of the size of a bean, deeply set in the skin, having a bright red color which at the first is dissipated by pressure, but becoming deeper, more sombre and permanent in color later on. They increase peripherally quite rapidly, so that within a week or ten days they may attain an area of an inch or an inch and a half. In general a goodly number of tumors appear scattered symmetrically over the whole body. As they grow they are followed by new ones which come along with greater or less rapidity in proportion as internal medication is pushed. If appropriate treatment be instituted, the first crop may be the only one. Unaffected by medicine their evolution continues, and in a fortnight the arms, foreams, perhaps the scapular region, not infrequently the back and anterior surface of the trunk, the gluteal regions, thighs and legs are invaded by these tumors.

The course of these gummata is, in general, quite regular and not subject to great variation. When developed they present a quite firm sensation, and this may be termed the period of condensation. As they grow older the red color becomes rather coppery, and while the periphery of the tumor may or may not seem firm, the central portions appear softer to the touch, conveying the impression that the tissues are permeated with a thick fluid. This we may denominate the stage of softening. In the majority of the cases there is no abscess formation, but rather a liquefaction of the gummy infiltration, which is, in general, promptly absorbed. The time occupied in the full development of these tumors is usually from ten days to two weeks, and after that their period of duration is variable.

In the ulcerative variety, the stage of condensation is very short, and softening in a marked degree is observed in a few days. The centre of the tumors assumes a dark red color in one or in several spots, and a sensation of fluid under the epidermis is distinctly made out. Then slight ulceration may occur in spots, often at the openings of the hair and sebaceous follicles, and very soon the epidermal roof the tumor melts away, and we soon see the gummatous ulcer with its slightly thickened, reddened, undermined, and perhaps everted edges, and its floor of a greenish-red, bathed in an unhealthy sanious pus.

In the *localized* form, the clinical history is similar to that preceding. The clinical history of the *neurotic* form has an individuality of its own. In the very early months of the diathesis, either in the stationary period of an early syphilide or at its decline, generally preceded or accompanied by severe neuralgic symptoms involving any cutaneous nerve, by severe sephalalgia, continuous or nocturnal; by rheumatoid pains in muscles or joints, and by general malaise and debility, this eruption makes its appearance with more or less promptitude and develops quite rapidly. In some instances the invasion is very acute, so that at the end of a week we may find fully developed tumors an inch or two long; in others and in the majority of instances the development is slower, and nearly two weeks elapse. Besides the general neuralgic symptoms, local pains on the site of the lesions or in the whole territory on which they are developed are experienced. These may be continuous or intermittent, and in some cases are as excruciating as in severe herpes zoster.

The eruption consists of two orders of lesions: first, tumors or nodosities seated in the subcutaneous tissue, and freely movable under the skin and over the fasciæ, though as they increase they may contract adhesions on both surfaces; second, oval or round tumors, or irregular plaques from fusion of tumors. The appearance and cause of the tumors are fully described.

In all cases of precocious gummata, the use of iodide of potassium is indicated, either combined with a mercurial or with the use of inunctions of mercurial ointment.

THE PUPIL IN ITS SEMEIOLOGICAL ASPECT.—Many observations have been made, from numerous standpoints, regarding pupillary conditions, yet with a few notable exceptions they have been studied in an isolated manner, relative to the particular disease or lesion of which they might be more or less symptomatic. They have been often looked upon, and are still regarded by some, as curious, interesting, but erratic phenomena, far too variable to be depended upon, and without any connecting thread upon which these conditions, as seen in a variety of diseases, could be strung. Few attempts have been made to grasp pupillary manifestations as a whole, still fewer to reduce the varying phenomena to principles or to reduce the laws by which they are controlled.

In an interesting paper in the July number of *The American Journal of the Medical Sciences*, Mr. William Macewen, of Glasgow, gives a brief outline of the physiological phenomenon pertaining to the movements of the pupil and then presents a series of personal observations.

He cites evidence to show that the suspension or abolition of cerebral functions in the living body is attended by mydriasis, the latter being the sequent of the former. If inquiry be made concerning the mechanism inducing this pupillary effect coincident with the arrest of cerebral function, the theory which explains the greater part, if not the whole of the phenomena, is that which has been so ably advocated by Mosso. The passive movements of the pupil are regulated by the vascular system of the iris, which is in complete harmony with that of the encephalon. In these conditions inducing general suspension of the cerebral function, a state of ischæmia prevails in the brain and iris inducing mydriasis. This likewise obtains in unilateral lesions, where the pressure is so great as to induce anæmia of brain and iris. Myosis may also be brought about by a like mechanism acting in the opposite direction. The "irritation" setting up congestion of the cerebral and meningeal vessels leads to congestion of the vessels of the iris, and so produces contraction of the pupil.

Dr. Macewen shows that when the function of the brain is in abeyance the pupils are in a state of stabile mydriasis.

When the function of the brain is interfered with by conditions usually included under the term "irritation," the pupils are in a state of myosis, sometimes labile, but generally stabile myosis.

The same pathological factors which cause myosis may also cause mydriasis, the degree in which these factors are present being the determining point between the former and the latter, and not merely the particular locus in the brain.

When the function of one-half of the cerebrum is placed in abeyance by a superficial or cortical lesion, the pupil on the same side as the lesion is in a state of stabile mydriasis.

When the function of one-half of the cerebrum is interfered with by some source of cortical irritation, the pupil on the corresponding side to the lesion is in a state of myosis.

Hemorrhage into the pons Varolii when strong causes strongly contracted pupils; but when it is more extensive, involving the gray matter beneath the aqueduct of Sylvius, a state of stabile mydriasis is induced.

Dr. Macewen concludes by pointing out the conditions under which myosis and mydriasis occur.

A Newspaper in Illinois recently brought suit against forty-three men who would not pay their subscriptions, and obtained judgment in each for the full amount of the claim. Of these, twenty-eight men made affidavit that they owned no more property than the law allowed them, thus preventing attachments. Then they, under the decision of the Supreme Court, were arrested for petty larceny, and bound over in the sum of $300 each. All but six gave bonds, while six went to jail. The postal law makes it larceny to take a newspaper and refuse to pay for it.—*Ex.*

